# Three-Dimensional Computed Tomography Metrics Reveal Divergent Functional–Structural Adaptation After Lobectomy in Chronic Obstructive Pulmonary Disease

**DOI:** 10.1093/icvts/ivag122

**Published:** 2026-04-21

**Authors:** Neeraj Singh, Monika Srivastav

**Affiliations:** Dr. D. Y. Patil Medical College Hospital and Research Centre, Dr. D. Y. Patil Vidyapeeth (Deemed-to-be-University), Pune, 411018 Maharashtra, India; Dr. D. Y. Patil Dental College and Hospital, Dr. D. Y. Patil Vidyapeeth (Deemed-to-be-University), Pune 411018, Maharashtra, India

Dear Editor,

We read with great interest the quantitative analysis by Kuroda et al., evaluating postoperative functional preservation and structural remodelling using 3D-CT cluster metrics after lobectomy.^1^ The integration of measured-to-predicted FEV1.0 ratio (MPFR), D-value, and low-attenuation area (LAA) provides a technically sophisticated framework. Several dataset-anchored considerations, however, further refine the clinical interpretation of these signals.

A primary interpretive inflection concerns the physiological meaning of the elevated MPFR in the COPD cohort (117.9% vs 110.7%). Because predicted postoperative FEV1.0 was derived from CT volumetry, the MPFR is intrinsically sensitive to baseline hyperinflation geometry. In lungs with heterogeneous emphysema, volumetric subtraction may systematically underestimate the functional contribution of preserved segments, thereby inflating the measured-to-predicted ratio independent of true postoperative gain.^2^ The observation that whole-lung *D*-value was substantially lower in the COPD group at baseline (1.36 vs 1.57) supports the presence of advanced structural heterogeneity that could bias volumetric prediction. From a perioperative decision standpoint, this raises the possibility that MPFR superiority partly reflects prediction model behaviour rather than purely biological volume-reduction benefit.

Second, the dissociation between stable %*D*-value (∼99% in both groups) and the marked %LAA expansion in non-COPD patients (134.9%) merits deeper mechanistic framing. *D*-value captures cluster size distribution topology, whereas LAA is highly sensitive to global lung inflation state.^3^ The concurrent stability of *D*-value suggests preserved microarchitectural complexity despite macroscopic low-density expansion. Clinically, this pattern is more consistent with postoperative mechanical overdistension than with structural parenchymal injury. This distinction is important because risk stratification models that treat LAA increase as emphysema progression could misclassify otherwise physiologically compensated patients.

The subgroup analysis based on emphysematous versus non-emphysematous resected lobes provides an additional nuance. Despite clear structural gradients, MPFR remained tightly clustered (∼113%-114%). This compression of functional dispersion suggests that global lung mechanics and diaphragmatic adaptation may dominate postoperative performance more than regional emphysema topology.^4^ For surgical planning, the data implicitly support a shift from lobe-centric emphysema mapping towards whole-lung functional reserve modelling.

The multivariable model is directionally reassuring, with COPD remaining independently associated with higher MPFR after adjustment. However, the covariate set is relatively parsimonious. Given the between-group differences in smoking burden, age, and baseline spirometry, residual physiological confounding, particularly dynamic hyperinflation and small airway disease, likely persists. Incorporating baseline lung volumes or inspiratory capacity in future models could sharpen causal attribution.^5^

Finally, the narrow spread of %*D*-value change across all strata suggests potential ceiling effects in cluster-based complexity metrics over a 6-month horizon. Whether longer-interval imaging would unmask delayed microstructural remodelling remains an open and clinically relevant question.

Overall, this well-executed study advances CT-based phenotyping of post-lobectomy adaptation. Future work integrating volumetric mechanics, dynamic physiology, and longitudinal structural trajectories may further operationalize these promising imaging biomarkers for precision surgical selection.

## FUNDING

No funding was received for this study.

## CONFLICT OF INTERESTS

The authors declare no conflict of interests relevant to this study.

## ETHICAL APPROVAL

Not required.

## DECLARATION OF GENERATIVE AI AND AI-ASSISTED TECHNOLOGIES IN THE MANUSCRIPT PREPARATION PROCESS

During the preparation of this work, the author(s) used ChatGPT and Grammarly to improve language clarity and grammatical accuracy. After using these tools, the author(s) reviewed and edited the content as needed and take full responsibility for the content of the publication.

## Data Availability

Not applicable, as no data were generated or analyzed in this study.
